# Expression and Functional Role of TRPV4 in Bone Marrow-Derived CD11c^+^ Cells

**DOI:** 10.3390/ijms20143378

**Published:** 2019-07-10

**Authors:** Robbe Naert, Alejandro López-Requena, Thomas Voets, Karel Talavera, Yeranddy A. Alpizar

**Affiliations:** 1 Laboratory of Ion Channel Research, Dept. of Cellular and Molecular Medicine, KU Leuven, 3000 Leuven, Belgium; 2 VIB Center for Brain & Disease Research, 3000 Leuven, Belgium

**Keywords:** Dendritic cells, TRPV4, TRP channels, phagocytosis

## Abstract

The increase in cytosolic Ca^2+^ is essential in key effector functions of dendritic cells (DCs), including differentiation, maturation, cytokine expression, and phagocytosis. Although several Ca^2+^-permeable ion channels have been described in DCs, the contribution of transient receptor potential (TRP) channels remains poorly understood. Here, we investigated whether TRPV4 plays a role in the differentiation, maturation, and phagocytosis of granulocyte-macrophage colony-stimulating factor (GM-CSF)-induced mouse bone marrow-derived cells (BMDCs). Using intracellular Ca^2+^ imaging experiments, we found that TRPV4 was functionally expressed in the plasma membrane of immature CD11c^+^ BMDCs and that its activity and expression were downregulated in CD11c^+^ BMDCs matured with lipopolysaccharide (LPS). Comparative analysis of the GM-CSF-stimulated cells showed that *Trpv4* knockout and wild-type bone marrow cultures had a similar distribution of differentiated cells, generating a heterogenous culture population rich in CD11c^+^, CD11b^+^ cells, and low levels of F4/80^+^ cells. The lack of TRPV4 did not prevent the LPS-induced nuclear translocation of NF-κB, the upregulation of the proinflammatory cytokines IL-6 and IL-12, or the upregulation of the maturation markers CD40, CD80, and CD86. In contrast, TRPV4-deficient CD11c^+^ BMDCs exhibited a significantly reduced endocytic capacity of IgG-coated beads, but the internalization of uncoated beads in the absence of TRPV4 was not affected. Taken together, our results demonstrate that TRPV4 was dispensable in the differentiation and maturation of mouse CD11c^+^ BMDCs but contributed to the mechanism underlying Fc receptor-mediated phagocytosis. Overall, our results further strengthen the role of TRPV4 in immune-related processes.

## 1. Introduction

Dendritic cells (DCs) are endowed with a remarkable plasticity that allows for complex genetic and phenotypic reprogramming in response to potentially harmful signals. These processes are generally initiated by the activation of pattern recognition receptors (e.g., toll-like receptors) in resting immature DCs, leading to mature DCs with upregulated antigen-presenting and costimulatory molecules, proinflammatory cytokines, and decreased endocytic activity [1]. These events are tightly regulated by cytosolic Ca^2+^ signals, which are orchestrated by diverse Ca^2+^-permeable ion channels, including Ca^2+^ release-activated Ca^2+^ channels (CRACs), L-type Ca^2+^ channels, purinergic receptors, and transient receptor potential (TRP) channels [2].

In DCs, CRAC channels are activated downstream of a signaling cascade involving the activation of phospholipase C (PLC)-γ2, increased levels of 1,4,5-triphosphate (IP_3_), and ensuing Ca^2+^ release from internal stores [3]. The latter decreases endoplasmic reticulum (ER) Ca^2+^ concentrations, inducing the opening of store-operated CRAC channels in the plasma membrane via the activation of stromal interaction molecule (STIM)1 and STIM2 [4,5,6]. In the presence of CRAC channel blockers or nonselective Ca^2+^ channel inhibitors, DCs fail to fully transition toward a mature phenotype, with lower expression of MHC-II and decreased production of proinflammatory cytokines [7]. However, DCs with impaired store-operated Ca^2+^ entry (SOCE) due to conditional deletion of STIM1/2 do not display abnormal in vitro differentiation, cytokine production, or phagocytosis [8], indicating that intracellular Ca^2+^ signaling, but not SOCE, through CRAC channels is required for the effector functions in DCs.

Several members of the TRP superfamily of cation channels have been described in DCs. Among these, TRPM2 is preferentially expressed in the lysosomal membranes as a Ca^2+^ release pathway in response to intracellular ADPR or external chemokine stimulation. TRPM2-lacking DCs exhibit deficient maturation and fail to migrate to the infection sites due to impaired upregulation of CXCR4, CCR5, and CCR7 chemokine receptors [9]. Another study has reported that *Trpv2* gene silencing in human monocyte-derived immature DCs prevented a reduction in fluid-phase endocytic capacity (pinocytosis) after a brief heat shock (up to 43 °C). Currents recorded at the shock temperature were abrogated by ruthenium red and in TRPV2-knockdown cells [10], suggesting the functional expression of this channel. TRPV1 and TRPV4 channels were also found in human monocyte-derived DCs, but unlike TRPV2, the pharmacological or genetic inhibition of these channels did not prevent a reduction in pinocytosis [10]. However, pinocytosis constitutes only one of the multiple endocytic mechanisms operating in DCs, which also include phagocytosis and receptor-mediated endocytosis.

Interestingly, TRPV4 is required for both the phagocytosis of nonopsonized particles and the uptake of IgG-coated beads through receptor-mediated mechanisms operating in macrophages [11]. However, whether TRPV4-mediated Ca^2+^ increase plays a similar role in the activation processes of DCs remains yet to be elucidated. In this paper, we show that TRPV4 was dispensable in the in vitro differentiation and TLR1/TLR2/TLR4-induced maturation of bone marrow-derived cells (BMDCs). Importantly, we identified a critical role for TRPV4 in Fc receptor (FcR)-mediated, but not in receptor-independent, endocytosis.

## 2. Results

### 2.1. TRPV4 Was Functionally Expressed in CD11c^+^ BMDCs

**Figure 1:**
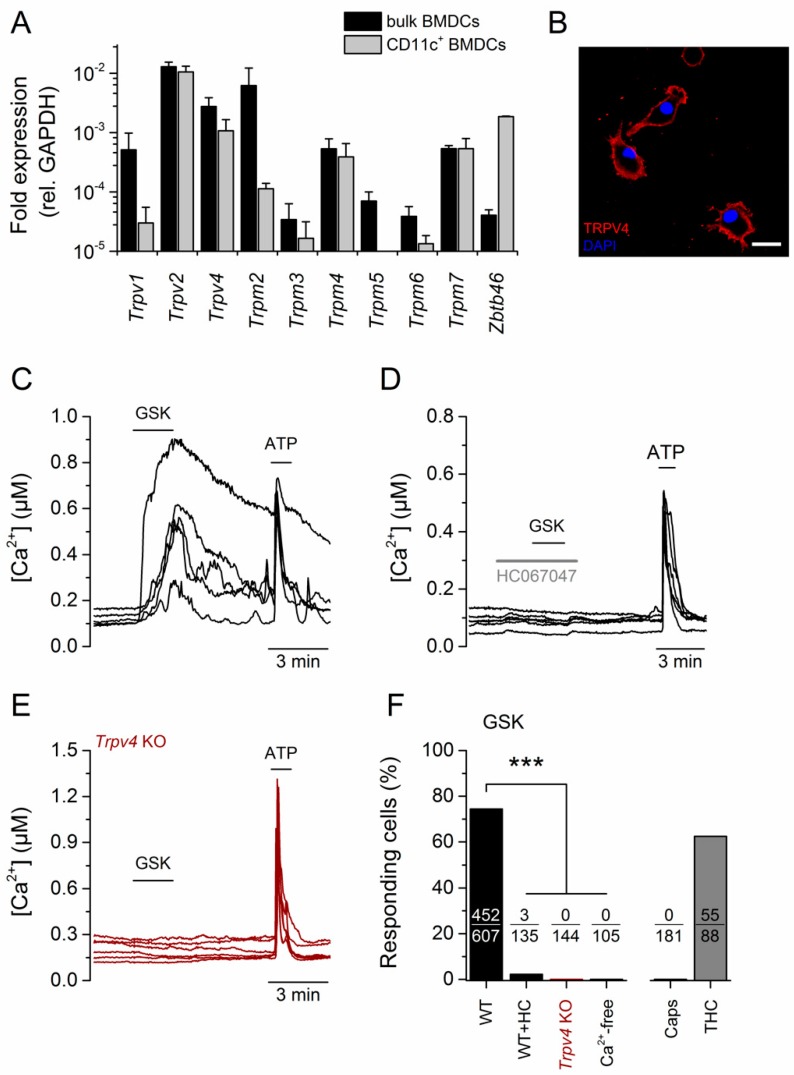
TRPV4 was functionally expressed in CD11c^+^ bone marrow-derived cells (BMDCs). (**A**) Expression profile of selected *Trp* genes in the total granulocyte-macrophage colony-stimulating (GM-CSF)-differentiated bone marrow-derived cell population (black bars) and in CD11c^+^-purified BMDCs (light gray). Values are relative to GAPDH expression. (**B**) Confocal image of CD11c^+^ BMDCs stained with an anti-TRPV4 antibody (red). The blue color corresponds to nuclear staining with DAPI. (**C–E**) Representative traces of intracellular Ca^2+^ concentration in CD11c^+^ BMDCs showing the effects of 300 nM of GSK1016790A (GSK). ATP (100 μM) was used as a positive control for intracellular Ca^2+^ increase. The TRPV4 antagonist HC067047 was used at 10 μM. (**F**) Percentage of CD11c^+^ BMDCs responding to the indicated stimulus. GSK, GSK1016790A (300 nM); HC, HC067047 (1 µM); Ca^2+^-free, Krebs with nominal [Ca^2+^] supplemented with 2.5 mM EDTA; Caps, Capsaicin (1 nM); THC, trans-Δ^9^-tetrahydrocannabinol (10 µM). The responding fraction is indicated within each bar. ***, *p* < 0.001, Fisher’s exact test.

We assessed the expression of *Trp* gene transcripts in BMDCs obtained after six days of culture in differentiation medium containing the granulocyte-macrophage colony-stimulating factor (GM-CSF, 20 ng/mL). Here, mRNA of *Trpv2*, *Trpv4*, and *Trpm2* was prominently expressed in the total BMDC population. *Trpv1*, *Trpm3*, *Trpm4*, *Trpm5*, and *Trpm7* were also detected, but at lower levels (black bars, Figure 1A). *Trpm1*, *Trpm8*, *Trpa1*, and the members of the TRPC channel subfamily were not detected after 40 cycles of amplification (data not shown). GM-CSF-derived cultures were enriched in CD11c^+^ cells but still contained a heterogeneous group of undifferentiated granulocytes that were separated using CD11c magnetic beads (>90% of CD11c^+^ cells, Supplementary Figure S1). In addition, mRNA analysis in the enriched population confirmed that *Trpv2*, *Trpv4*, *Trpm4*, and *Trpm7* were highly expressed in CD11c^+^ cells. In contrast, *Trpv1*, *Trpm2*, *Trpm5*, and *Trpm6* transcript levels were significantly reduced in the CD11c^+^ BMDC population (gray bars, Figure 1A), indicating that these were mainly expressed in CD11c^-^ nonselected cells. The increased expression of the CD11c^+^-specific marker *Zbtb46* [12] further confirmed the enrichment in CD11c^+^ cells.

Similarly to what has been reported in monocyte-derived human DCs [10,13], we found that TRPV2 was functionally expressed in mouse CD11c^+^ BMDCs. Indeed, ratiometric Ca^2+^ imaging experiments showed that more than 60% (55/88) of these cells responded to 50 µM of the TRPV2 agonist trans-Δ^9^-tetrahydrocannabinol (THC, Supplementary Figure S2A, Figure 1F). In contrast, application of the TRPV1 agonist capsaicin (1 µM) did not evoke an increase in intracellular Ca^2+^ concentration in CD11c^+^ BMDCs (0/181, Supplementary Figure S2B, Figure 1F). Together with the low levels of mRNA in the CD11c^+^ BMDCs (Figure 1A), our data indicated that TRPV1 was not functionally expressed in the plasma membrane of these cells.

Using a validated antibody against TRPV4 (Supplementary Figure S3), we found that TRPV4 was predominantly expressed in the plasma membrane of CD11c^+^ cells (Figure 1B). We confirmed the functional expression of the channel in ratiometric Ca^2+^ imaging experiments, in which 74.5% (452/607) of the CD11c^+^ BMDCs responded to 300 nM of the TRPV4 agonist GSK1016790A [14] (hereafter referred to as GSK, Figure 1C,F). Cells were virtually unresponsive to GSK in the presence of the TRPV4 agonist HC067047 (2.2%, 3/135, Figure 1D,F) or in the absence of extracellular Ca^2+^ (0/105, Supplementary Figure S2C, Figure 1F). Likewise, CD11c^+^ BMDCs from *Trpv4* knockout (KO) mice were unresponsive to 300 nM of GSK (0/144, Figure 1E,F).

### 2.2. Differentiation of BMDCs Was Independent of TRPV4

**Figure 2:**
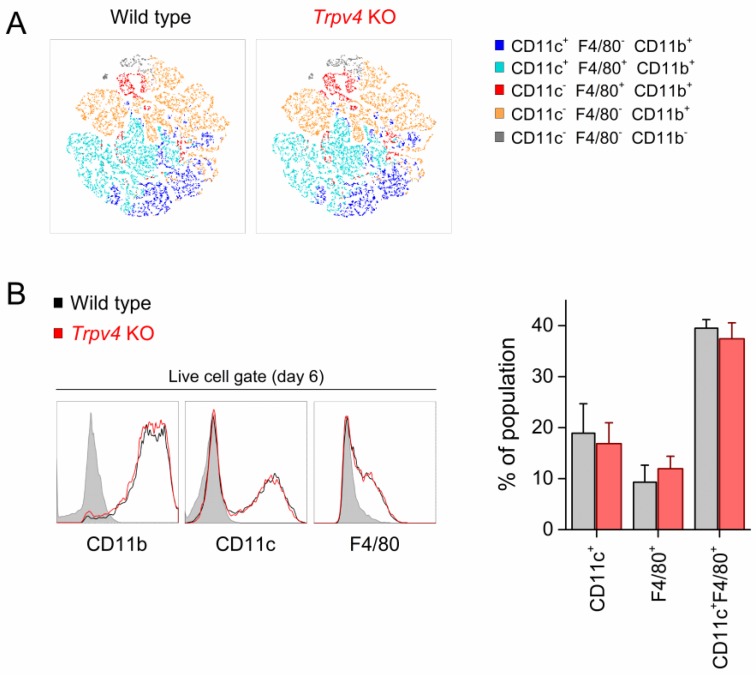
TRPV4 was dispensable in the differentiation of CD11c^+^ BMDCs. (**A**) Color-coded two-dimensional t-distributed stochastic neighbor embedding (tSNE) representations of the total bone marrow-derived cell population (20,000 cells) defined by the surface markers CD11b, CD11c, and F4/80. (**B**) Histograms showing surface expression of the indicated markers in bone marrow-derived cells from wild-type (WT, black traces) and *Trpv4* knockout (KO, red traces) mice. The shaded histograms represent specificity (fluorescence minus one) controls. The bar graph shows the percentage of different cell populations present in total bone marrow-derived cell cultures defined by the surface expression of CD11b, CD11c, and F4/80. The data are represented as mean ± SEM of nine independent experiments.

Next, we investigated whether TRPV4 was required for bone marrow (BM) cell differentiation. For this, we compared the composition of the populations of wild-type (WT) and *Trpv4* KO BM cells after six days of incubation with GM-CSF. We used the CD11b surface marker to quantify total granulocytes and F4/80 and CD11c to distinguish between macrophage-like and DC-like populations. In addition, t-distributed stochastic neighbor embedding (tSNE) analysis of flow cytometry data (20,000 cells per genotype) and analysis of individually gated populations showed that WT- and *Trpv4* KO-derived cells displayed similar population heterogeneity after differentiation (Figure 2A,B). This mainly consisted of CD11c F4/80 double-positive cells and lower proportions of cells expressing only CD11c or F4/80. Likewise, WT and TRPV4-deficient BM-derived cells exhibited similar levels of expression of these surface markers (Figure 2B). TRPV4-deficient cells had similar expressions of *Trpv2*, *Trpm4*, and *Trpm7* genes and significantly lower expression of *Trpm2* (*p* = 0.048, two-tailed, unpaired *t*-test) compared to WT cells (Supplementary Figure S4). Although TRPM2 is involved in the differentiation of BMDCs [9], based on the tSNE distribution, we could not observe differences in the differentiated populations (Figure 2A). This excluded an indirect effect on differentiation due to TRPM2 downregulation.

### 2.3. TRPV4 Was Downregulated in Mature BMDCs

**Figure 3:**
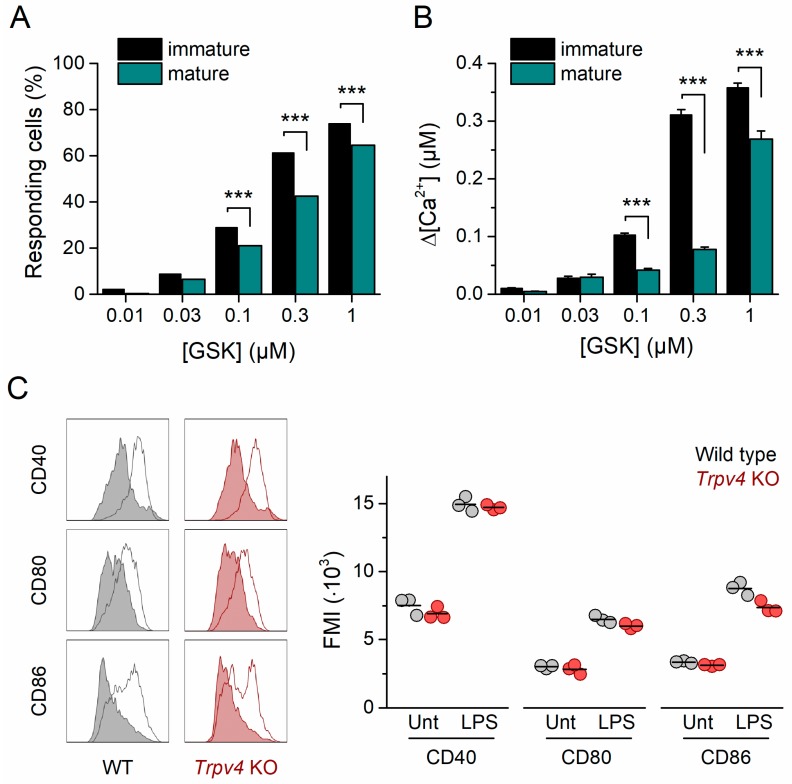
TRPV4 was downregulated in mature CD11c^+^ BMDCs. (**A**) Concentration dependence of immature (black bars) and mature (dark cyan bars) CD11c^+^ BMDC responding fraction. ***, *p* < 0.001, Fisher’s exact test. (**B**) Average amplitude of the responses of immature (black) and mature (dark cyan) CD11c^+^ BMDCs to GSK1016790A applied at various concentrations (*n* > 300 per data point). ***, *p* < 0.0001, two-tailed unpaired *t*-test. (**C**) Representative histograms of WT and *Trpv4* KO CD11c^+^ BMDCs untreated (shaded histogram) or treated (empty histogram) with lipopolysaccharide (LPS). Cells were harvested after six days of differentiation and incubated with 100 ng/mL of LPS for 24 h. Histograms correspond to living, single cells gated within the CD11c^+^ population. The right panel shows corresponding mean fluorescence intensity (MFI) values for CD40, CD80, and CD86 expression in WT and *Trpv4* KO BMDCs untreated or treated with 100 ng/mL of LPS for 24 h. Data points correspond to living, single cells gated within the CD11c^+^ population. The horizontal bar represents the mean of three independent experiments. Unt, untreated cells. LPS, LPS-treated cells.

To determine whether the TRPV4 expression level was dependent on the activation state of the BMDCs, we incubated the cells with lipopolysaccharide (LPS) from *Escherichia coli* (100 ng/mL) and characterized the intracellular Ca^2+^ response to varying doses of GSK. Notably, the mature CD11c^+^ BMDC (mBMDC) population exhibited a significantly lower percentage of responding cells (Figure 3A) and a maximal increase in intracellular Ca^2+^ concentration (Figure 3A,B) with respect to immature CD11c^+^ BMDCs (iBMDCs). These results suggest that TRPV4 expression levels decrease upon CD11c^+^ BMDC maturation. Indeed, we found that *Trpv4* transcripts in mBMDCs were significantly lower than in iBMDCs (*p* = 0.034, two-tailed, unpaired *t*-test, Supplementary Figure S5).

The recognition of LPS by TLR4 is followed by the endocytosis of the TLR4 signaling complex, a Ca^2+^-dependent process that constitutes a crucial early event in the responses of macrophages to LPS [15]. Thus, we sought to determine whether TRPV4 is required for the activation and transition of iBMDCs to mature cells. For this, we quantified the upregulation of the costimulatory molecules CD40, CD80, and CD86 in LPS-treated *Trpv4* KO iBMDCs. Similarly to WT cells, unstimulated *Trpv4* KO CD11c^+^ BMDCs contained a low percentage of spontaneously matured cells, which were characterized by a high expression of CD40, CD80, and CD86 (Figure 3C). Incubation with LPS induced an upregulation of these maturation markers in *Trpv4* KO CD11c^+^ iBMDCs. The expression levels of the maturation markers were similar to those measured in WT CD11c^+^ mBMDCs (Figure 3C), indicating that TRPV4 was not required for the acquisition of the mature phenotype after TLR4 activation. Interestingly, incubation with the polypeptide Pam3CSK4, a potent TLR1/TLR2 agonist, also induced the upregulation of CD40, CD80, and CD86 in *Trpv4* KO cells to levels similar to those found in WT CD11c^+^ BMDCs. Altogether, these results indicate that TLR1/TLR2/TLR4-induced maturation signaling was not defective in TRPV4-deficient cells (Supplementary Figure S6).

### 2.4. NF-κB Nuclear Translocation and Inflammatory Gene Expression Was Unaltered in Trpv4 KO CD11c^+^ BMDCs

Next, we sought to determine whether TRPV4 contributes to the LPS-induced translocation of NF-κB to the nucleus and the expression of proinflammatory cytokines. For this, we incubated iBMDCs with LPS and quantified the subcellular distribution of NF-κB. We examined NF-κB protein in whole-cell lysates after subcellular fractioning. Immature WT and *Trpv4* KO cells featured a high content of NF-κB in the cytoplasmic fraction, whereas it was undetectable in the nuclear fraction. In cells of both genotypes, the pool of NF-κB was reduced in the cytoplasm and increased in the nuclear fraction after 30 min incubation with LPS (Supplementary Figure S7).

**Figure 4:**
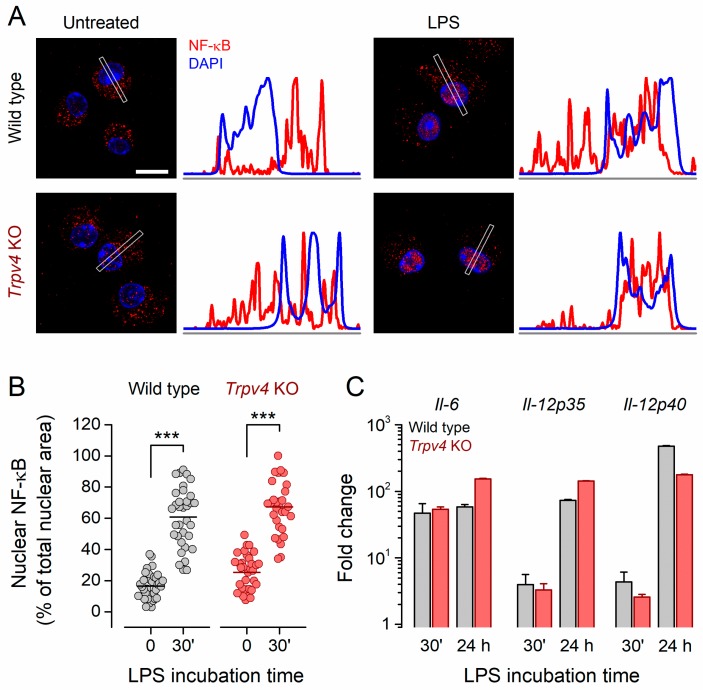
LPS-induced cytokine production occurred independently of TRPV4. (**A**) Representative confocal immunofluorescence microscopy images of fixed BMDCs untreated or treated with LPS (100 ng/mL). Cell stainings correspond to NF-κB p65 (red) and DAPI (nuclear, blue). Scale bar, 10 µm. The average linear intensity along the gray rectangle is represented next to the corresponding image. (**B**) Percentage of the total nuclear area stained by NF-κB p65 staining. The horizontal bar represents the mean. ***, *p* < 0.001, Tukey’s multiple comparison test. (**C**) Relative expression of cytokines in WT and *Trpv4* KO BMDCs after 30 min and 24 h incubation with 100 ng/mL of LPS. Expression levels were normalized to GAPDH and the genotype-matched untreated samples. The bars represent the mean ± SEM of three independent experiments.

Confocal images of unstimulated BMDCs from both WT and *Trpv4* KO mice confirmed that these cells exhibited a scattered distribution of NF-κB throughout the cytoplasm and low expression in the nucleus (Figure 4A,B; Supplementary Figure S8). Incubation with LPS significantly increased nuclear NF-κB in WT iBMDCs (from 17.6% ± 1.2% to 60.9% ± 3.2%, *p* < 0.001, two-tailed Fisher’s test). LPS induced a similar shift of the NF-κB distribution in *Trpv4* KO BMDCs (Figure 4B, from 25.3% ± 2.0% to 67.3% ± 3.1%, *p* < 0.001, two-tailed Fisher’s test), indicating that TRPV4 was not necessary for the mobilization of this transcription factor to the nucleus. Consequently, the expression of proinflammatory cytokines was similar between WT and *Trpv4* KO BMDCs (Figure 4C).

### 2.5. TRPV4 Contributed to the Receptor-Mediated Phagocytosis of Immature CD11c^+^ BMDCs

**Figure 5:**
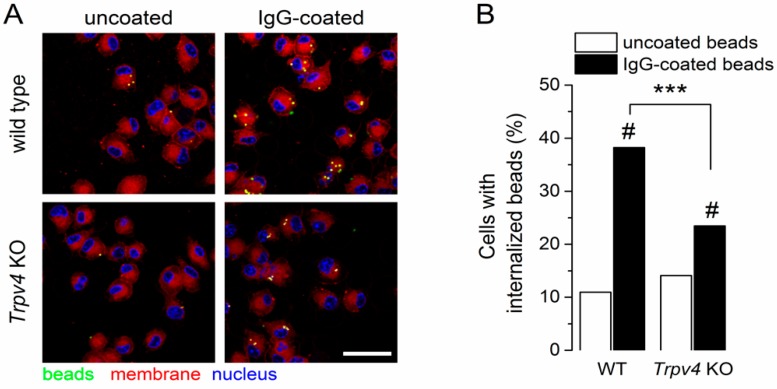
TRPV4-deficient BMDCs exhibited impaired FcR-dependent phagocytosis. (**A**) Representative confocal images of wild-type and *Trpv4* KO BMDCs after treatment with uncoated or IgG-coated fluorescent microspheres. Scale bar, 20 µm. (**B**) Percentage of cells with internalized beads. Data were collected from 10 randomly selected fields per condition from three independent experiments. ***, *p* < 0.001, Fisher’s exact test for the same treatment condition across phenotypes. #, *p* < 0.001, Fisher’s exact test between different treatment conditions within the same phenotype.

Since intracellular Ca^2+^ signals are required for both Fc receptor-dependent and receptor-independent endocytic events in BMDCs [8], we wondered whether TRPV4 contributes to these processes. To determine whether TRPV4 plays a role in FcR-independent endocytosis, we incubated WT and *Trpv4* KO BMDCs with uncoated fluorescence latex beads for 30 min and quantified the number of cells containing beads. We found a similar proportion of WT and *Trpv4* KO BMDCs with internalized beads (Figure 5A,B), suggesting that TRPV4 is not required for FcR-independent phagocytosis.

Next, we used IgG-coated beads to evaluate the role of TRPV4 in FcR-dependent endocytosis. For both WT and *Trpv4* KO BMDCs, the uptake of IgG-coated beads was significantly increased compared to uncoated beads (Figure 5B). However, BMDCs deficient in TRPV4 exhibited a significantly lower proportion of bead-containing cells (38.2% vs. 23.4%, *p* < 0.05, two-tailed Fisher’s test). Altogether, these results demonstrate that TRPV4 was dispensable in the FcR-independent process, but contributed to FcR-mediated phagocytosis.

## 3. Discussion

Physiological processes during the lifespan of DCs, such as differentiation, phagocytosis, maturation, and cytokine expression, are regulated by changes in cytosolic Ca^2+^ concentrations [2]. Whereas store-operated Ca^2+^ entry mechanisms, ryanodine receptors, L-type Ca^2+^ channels, and purinergic receptors have been implicated in these events, the role of TRP channels has remained poorly defined. Here, we showed that the Ca^2+^-permeable channel TRPV4 was functionally expressed in CD11c^+^ BMDCs. We found that TRPV4 was downregulated in mature BMDCs and was implicated in receptor-mediated phagocytosis but played no significant role in the differentiation or maturation of BMDCs.

In this study, we focused on loosely adherent CD11c^+^ populations, as these are widely used as a cellular model for DCs. However, these cultures comprise a heterogenous population of CD11b^+^ cells with phenotypic and genotypic signatures of DCs, macrophages [16], and neutrophils [17,18]. For the purpose of our study, an in-depth phenotypic classification in terms of MHC-II, CD115, CD123, and CD135 [16] expression levels was not necessary, as the purified CD11c^+^ displayed the main features of antigen-presenting cells (e.g., maturation, phagocytosis, cytokine expression).

Our screening showed significant expression of *Trpv2*, *Trpv4*, *Trpm2*, *Trpm4*, and *Trpm7*. From these, TRPV2, TRPM2, and TRPM4 have been shown to contribute to DC differentiation, phagocytosis, maturation, and chemotactic events [9,10,19]. However, the role of TRPV4 has remained largely unexplored. Using BMDCs from *Trpv4* KO mice, we here demonstrated that TRPV4 was dispensable in the GM-CSF-induced differentiation of CD11c^+^ BMDCs. Phenotypically, TRPV4-deficient BMDCs committed to CD11c^+^F4/80^+^ and CD11c^+^F4/80^−^ phenotypes, as observed for WT cells. Likewise, TRPV4 was not required for the upregulation of surface markers associated with the mature phenotype or for the LPS-induced nuclear translocation of NF-κB and the ensuing production of proinflammatory cytokines.

In contrast, in the absence of functional TRPV4, BMDCs exhibited lower FcR-mediated endocytosis. Similar findings have been described in bone marrow-derived macrophages, in which the phagocytosis of IgG-coated beads was regulated by the cooperation of matrix stiffness-associated TRPV4 activation and TLR4-mediated signaling [11]. However, our experiments suggest that TRPV4 may also be activated during the phagocytic event in the absence of LPS and exogenous TRPV4 activators.

TRPV4 activation might occur during receptor-mediated endocytosis through a mechanism involving PLC activation and changes in membrane phosphoinositide content. As previously described for phagocytic cells, we hypothesize that after FcR ligation in CD11c^+^ BMDCs, tyrosine kinases of the Syk family activate PLC-γ [20,21,22], inducing the production of IP_3_ levels from membrane-anchored phosphatidylinositol 4,5-bisphosphate (PI(4,5)P_2_) [23]. In addition to activating Ca^2+^-release channels in the ER, the newly synthesized IP_3_ may sensitize TRPV4 to the mechanical stress produced in the vicinity of the engulfing membranes [24]. Furthermore, the depletion of PI(4,5)P_2_ might release the tonic inhibition of PI(4,5)P_2_ on TRPV4, resulting in increased channel activity [25,26] and the suppression of actin polymerization [27]. The latter allows for FcR clustering and membrane deformation [28], which could constitute the mechanical stimulus for the activation of TRPV4 [29]. Thus, TRPV4-mediated Ca^2+^ influx could further contribute to the actin depolymerization required for the initial formation of the phagosomal cup [30]. Our results also indicate that TRPV4 was recruited downstream of FcR ligation, as the phagocytosis of uncoated beads remained unaltered in *Trpv4* KO BMDCs.

Nonetheless, the fact that the absence of TRPV4 resulted in only a partial reduction of the phagocytosis of IgG-coated beads demonstrates that TRPV4 was not the only source for the spatially restricted Ca^2+^ increase that was required for this process. The related channel TRPV2, which we found to be also functionally expressed in BMDCs, may also contribute, similarly to what has been observed in peritoneal macrophages [31]. The selective downregulation of TRPV4, and not TRPV2, in mature CD11c^+^ BMDCs may explain their slower and less efficient particle uptake [32]. Determining whether TRPV2 and TRPV4 are activated through different pathways and/or whether they are required at different stages during the phagocytic events deserves further investigation.

We also identified TRPV1 transcripts, but in contrast to previous reports [33], we could not corroborate their functional expression in purified CD11c^+^ BMDCs, as evidenced by the absence of a detectable Ca^2+^ response to capsaicin. In addition, the purified CD11c^+^ BMDC population showed significantly lower expression of TRPV1 when compared to the total CD11b^+^ pool, indicating that undifferentiated CD11c^−^ (CD11b^+^ granulocytes) are richer in TRPV1 transcripts. It has been previously reported that TRPV1 is found in a small subpopulation of CD11c^+^F4/80^−^ freshly isolated splenocytes and that its expression varies across different mouse strains, the lowest being in C57BL/6J [34], the same background used in our study and elsewhere [33,35]. Thus, a deeper characterization will be required to identify the phenotypic signature of the TRPV1-expressing CD11c^+^ population. Overall, the discrepancies in the functional expression of TRPV1 in BMDCs [33,35] could also indicate that TRPV1 is under a regulatory mechanism that is sensitive to culture conditions, namely the concentration of GM-CSF, the addition of IL-4, the duration of the culture, etc., which may result in the generation of TRPV1-expressing cells. Likewise, we do not exclude that the functional expression of TRP channels might differ among the phenotypically different subpopulations of DCs found in vitro and more importantly in vivo.

In conclusion, our results show that TRPV4 was functionally expressed in bone marrow-derived CD11c^+^ cells and was required for FcR-mediated phagocytosis. Since we used in vitro-generated CD11c^+^ DC surrogates, it is of paramount importance that these findings are corroborated in different DC subsets in vivo. A DC-specific TRPV4 knockdown mouse model will be highly instrumental in this, allowing for the selective deletion of TRPV4 at specific time points during mouse development and immune challenges. Overall, our findings further strengthen the emerging concept of TRP channels as modulators of immune-related processes.

## 4. Materials and Methods

### 4.1. Cells and Transfection

HEK293T cells were grown in DMEM containing 10% (*v*/*v*) fetal calf serum, 4 mM of L-alanyl-L-glutamine, 100 U/mL of penicillin, and 100 μg/mL of streptomycin at 37 °C in a humidity-controlled incubator with 10% CO_2_. For confocal imaging, cells were transiently transfected with mouse TRPV4 cloned into the bicistronic pCAGGSM2-IRES-GFP vector [36] using TransIT-293 transfection reagent (Mirus, Madison, WI, USA).

### 4.2. Animals

*Trpv4* KO mice were kindly provided by Prof. W. Liedtke (Duke University, Durham, NC, USA). Mice were housed under identical conditions, with a maximum of four animals per cage, on a 12-h light–dark cycle and with food and water *ad libitum*. Ten–twelve-week-old male mice were used in all experiments. All animal experiments were carried out in accordance with the European Union Community Council guidelines and were approved by local ethics committees.

### 4.3. Isolation of Bone Marrow-Derived CD11c^+^ Cells

CD11c^+^ cells were derived from bone marrow as previously described [17]. Briefly, femurs and tibiae from CO_2_ euthanized mice were dissected and cleaned from the surrounding muscle tissue. Thereafter, intact bones were rinsed in 70% ethanol for 2 min for disinfection and washed with PBS. Bone ends were cut with scissors, and the marrow was flushed with RPMI-1640 medium using a syringe fit with a 26-G diameter needle. Clusters within the marrow suspension were disintegrated using vigorous pipetting. Erythrocytes were lysed in RBC Lysis Buffer solution (eBioscience, Vienna, Austria), and mononuclear cells were counted and cultured at 0.6 × 10^6^ cells per ml in RPMI-1640 medium supplemented with 10% FCS, 2 mM glutamine, 100 U/mL penicillin–streptomycin (Gibco, Aalst, Belgium), 50 µM β-mercaptoethanol, and 20 ng/mL GM-CSF (PeproTech, Rocky Hill, NJ, USA). Fresh medium was added on day 3 of culturing. For maturation experiments, cells were treated with LPS (100 ng/mL) or Pam3CSK4 (1 µg/mL) on day 6 (*E. coli* 0127:B8, Sigma-Aldrich, Overijse, Belgium). After treatment, cells were loosened using a cell scraper, and the CD11c^+^ population was purified using CD11c MicroBeads (Miltenyi Biotec, Leiden, the Netherlands) following the manufacturer’s instructions. CD11c^+^ population enrichment was systematically confirmed by FACS to be above 90% of the collected cells (Supplementary Figure S1).

### 4.4. RNA Isolation and qPCR

Total RNA from isolated CD11c^+^ cells was extracted using the RNeasy Mini Kit (Qiagen, Antwerp, Belgium) following the manufacturer’s protocols. In addition, cDNA was synthesized from 1 µg of total RNA using Ready-To-Go You-Prime First-Strand Beads (GE Healthcare, Diegem, Belgium). Quantitative PCR reactions (20 μL) containing 3 μL of cDNA template (diluted 1:5), Universal TaqMan MasterMix (2X concentrated, Thermo Fisher Scientific, Waltham, MA, USA), specific TaqMan probes (Table 1, 20X concentrated, Life Technologies), and H_2_O were performed with the 7500 Fast Real-Time PCR System (Thermo Fisher Scientific) using the following program: 50 °C for 2 min and 95 °C for 10 min, followed by 40 cycles of 95 °C for 15 s and 60 °C for 1 min. Nontemplate controls (NTCs) were used as negative controls in every experiment.

### 4.5. Confocal Imaging

Purified CD11c^+^ BMDCs were allowed to attach in glass coverslips for 24 h. Thereafter, cells were fixed with cold paraformaldehyde and permeabilized with 0.2% Triton X-100. Cells were blocked with antimouse CD16/32 polyclonal antibody (1 µg/mL, eBioscience) in 5% sheep serum (Sigma-Aldrich) for 3 h. After two rinsing steps with PBS, cells were incubated overnight at 4 °C with a rabbit anti-TRPV4 antibody (1:200, ACC-124, Alomone labs, Jerusalem, Israel). Cells were later incubated for 1 h with an anti-rabbit-Alexa 546 antibody (1:2000, Thermo Fisher Scientific). Coverslips were mounted on glass slides using DAPI-containing mounting solution (VectaShield, Vector Laboratories, Burlingame, CA, USA). The confocal images of labeled cells were collected using the optimal pinhole size for the 63X oil objective of a Zeiss LSM 510 Meta Multiphoton microscope (Carl Zeiss AG, Oberkochen, Germany). The specificity of the anti-TRPV4 antibody was confirmed in HEK293T cells transfected with mouse TRPV4/GFP bicistronic vector (Supplementary Figure S3).

The nuclear translocation of NF-κB was evaluated in BMDCs from wild-type and *Trpv4* KO mice exposed to LPS (100 ng/mL) for 30 min and 24 h. After treatment, cells were fixed and blocked as described above and incubated with a primary antibody against NF-κB (1:250; Cell Signaling Technology, Leiden, the Netherlands, #4764) overnight at 4 °C. This was followed by anti-rabbit-Alexa Fluor 633 (1:600; Invitrogen, Waltham, MA, USA, A21070) for 1 h at room temperature. Coverslips were mounted in glass slides using DAPI-containing mounting solution (VectaShield). Confocal images were obtained from 10 randomly selected fields from three independent experiments using the optimal pinhole size for the 63X oil objective on a Zeiss LSM 880 (Carl Zeiss AG). Nuclear NF-κB was quantified as the area occupied by the NF-κB signal relative to the total nuclear area (in pixels). The nuclear area was defined by DAPI staining. For quantification, *xy* images were acquired after quick *z* scanning, and the optimal *z* value was defined ad hoc, corresponding to the middle position of the majority of the nuclei in the *xy* plane.

### 4.6. Intracellular Ca^2+^ Imaging

Cells were incubated with Fura-2 acetoxymethyl ester for 30 min at 37 °C. For recordings, bath solutions prepared in Krebs (containing, in mM, 150 NaCl, 6 KCl, 1.5 CaCl_2_, 1 MgCl_2_, 10 HEPES, 10 glucose, and titrated to 7.4 with NaOH) were perfused by gravity via a multibarreled pipette tip. Intracellular Ca2+ concentration was monitored through the ratio of fluorescence measured upon alternating illumination at 340 and 380 nm using an Eclipse Ti (Nikon, Groot-Bijgaarden, Belgium) fluorescence microscopy system.

### 4.7. FACS

BMDC or CD11c^+^ purified populations were stained with antibodies against CD11b, CD11c, F4/80, CD40, CD80, and CD86 according to standard procedures (BD Biosciences, Erembodegem, Belgium). Cell viability was determined by staining with the Zombie Yellow Fixable viability kit (BioLegend, Antwerpen, Belgium). Percentages of labeled cells were determined by performing flow cytometry on a Canto II HTS cytometer (BD Biosciences) on at least 2 × 10^5^ cells. Data analysis was performed with FlowJo software (v10.5.3, FlowJo LLC, Ashland, OR, USA).

### 4.8. Nuclear Extraction and Western Blot

Cellular fractions were isolated using NE-PER Nuclear and Cytoplasmic Extraction Reagents (ThermoFisher Scientific; 78833) following the manufacturer’s protocols. Briefly, BMDCs were collected on ice and centrifuged at 500× *g* for 5 min. Cells were rinsed with cold PBS, transferred to a 1.5-mL tube, and centrifuged at 500× *g* for 3 min. The sample was resuspended in CER I buffer and incubated on ice for 10 min. CER II buffer was added, and cells were incubated for 1 min followed by centrifugation at 16,000× *g* for 5 min. The supernatant containing the cytosolic fraction was collected and stored on ice until use. The remaining cell pellet was resuspended in NER buffer and vortexed every 10 min for a total of 40 min. Afterwards, the lysates were centrifuged at 16,000× *g* for 10 min, and the supernatant containing the nuclear fraction was collected and stored on ice until use.

Protein content was determined via BCA assay following the manufacturer’s instructions. Nuclear and cytosolic fractions were mixed with 3X Laemmli Sample Buffer (Sigma-Aldrich) and were incubated at 95 °C for 5 min. Samples were cooled for 2 min on ice prior to SDS-PAGE analysis. Samples (10 µg) were loaded onto Mini-PROTEAN TGX Stain-Free Precast 4%–15% gel (Bio-Rad, Hercules, CA, USA) and electrophoretically separated at 200 V for 35 min. Next, the gel was transferred to a PVDF membrane using the Trans-Blot Turbo RTA Transfer Kit (Bio-Rad) and the Trans-Blot Turbo System (Bio-Rad). The membrane was washed in TBS-T for 10 min and blocked with TBS-T containing 5% dry milk powder for 2 h at RT. After rinsing, the membrane was cut and incubated with primary antibodies for NF-κB (1:1000, Cell Signaling Technologies, #4764), Na^+^/K^+^-ATPase (1:10,000, Abcam, Cambridge, UK, ab7671), GAPDH (1:5000, Sigma-Aldrich, G9545), and β-actin (1:10,000, Sigma-Aldrich, A1978) overnight at 4 °C. The next day, the membranes were rinsed and incubated with HRP-conjugated antirabbit or antimouse secondary antibodies (1:5000) for 1 h at room temperature. The blots were developed using the Clarity Western ECL Substrate (Bio-Rad) following the manufacturer’s protocol.

### 4.9. Phagocytosis Assay

One-micrometer Fluoresbrite yellow-green carboxylated latex microspheres (Polysciences, Inc., Hirschberg an der Bergstrasse, Germany) were incubated overnight with 1 mg/mL of mouse IgG (Jackson ImmunoResearch Laboratories, Ely, UK). After rinsing the unbound antibody, the microspheres were added to BMDCs cultured in glass coverslips at a concentration of 9.1 × 10^7^ beads/mL and incubated for 30 min in a humidified incubator at 37 °C and 5% CO_2_. Uncoated microspheres were used to determine FcR-independent phagocytosis. Unbound or attached noninternalized beads were washed in consecutive rinsing steps with cold PBS. BMDCs were then incubated with CellMask™ Deep Red Plasma Membrane Stain (ThermoFisher Scientific) for 2 min at room temperature, rinsed in cold PBS, fixed, and blocked as described above. Coverslips were mounted on glass slides using DAPI-containing mounting solution (VectaShield), and images were collected from 10 randomly selected fields using a Zeiss LSM 880 (Carl Zeiss AG) confocal microscope. During image acquisition, a quick *z* scan was performed to identify the optimal *z* value based on the middle position of the majority of the nuclei. This provided a *bona fide* landmark for the confocal images in the *xy* plane that included the cytoplasmic area and therefore the internalized beads (Supplementary Figure S9).

### 4.10. Data and Statistical Analysis

Confocal images were analyzed using Fiji software [37]. Data analysis was performed using Origin 9.0 (OriginLab Corporation, Northampton, MA, USA). Magnitudes were statistically compared using GraphPad Prism version 7.0d for MacOS (GraphPad Software, La Jolla, CA, USA, (www.graphpad.com)). Differences were considered to be statistically significant when *p* < 0.05. The specific statistical tests used for group comparison are indicated in the legends of the corresponding figures.

## Figures and Tables

**Table 1:** List of TaqMan probes.

Gene Name	Probe ID (in Applied Biosytems)
*Trpa1*	Mm00625268_m1
*Trpv1*	Mm01246302_m1
*Trpv2*	Mm00449223_m1
*Trpv3*	Mm00454996_m1
*Trpv4*	Mm00499025_m1
*Trpv5*	Mm01166037_m1
*Trpv6*	Mm00499069_m1
*Trpm1*	Mm00450619_m1
*Trpm2*	Mm00663098_m1
*Trpm3*	Mm00616485_m1
*Trpm4*	Mm00613173_m1
*Trpm5*	Mm01129032_m1
*Trpm6*	Mm00463112_m1
*Trpm7*	Mm00457998_m1
*Trpm8*	Mm00454566_m1
*Trpc1*	Mm00441975_m1
*Trpc2*	Mm00441984_m1
*Trpc3*	Mm0044690_m1
*Trpc4*	Mm00444284_m1
*Trpc5*	Mm00437183_m1
*Trpc6*	Mm01176083_m1
*Trpc7*	Mm00442606_m1
*Zbtb46*	Mm00511327_m1
*Il-6*	Mm00446190_m1
*Il-12a*	Mm00434169_m1
*Il-12b*	Mm01288989_m1
*Gapdh*	Mm99999915_g1

## Supplementary Materials

Supplementary materials can be found at https://www.mdpi.com/1422-0067/20/14/3378/s1.
