# Supplementary material for: Expression and Functional Role of TRPV4 in Bone Marrow-Derived CD11c+ Cells
Source: Int J Mol Sci. 2019 Jul 10;20(14):3378. doi: 10.3390/ijms20143378 (PMC6678969; doi:10.3390/ijms20143378)
Supplement: Supplementary file 1 [file ijms-20-03378-s001.pdf]

International Journal of *Molecular Sciences*

MDPI

**Supplementary Information**

**Expression and functional role of TRPV4 in bone marrow-derived CD11c+ cells.**

Robbe Naert, Alejandro López-Requena, Thomas Voets, Karel Talavera, Yeranddy A. Alpizar.

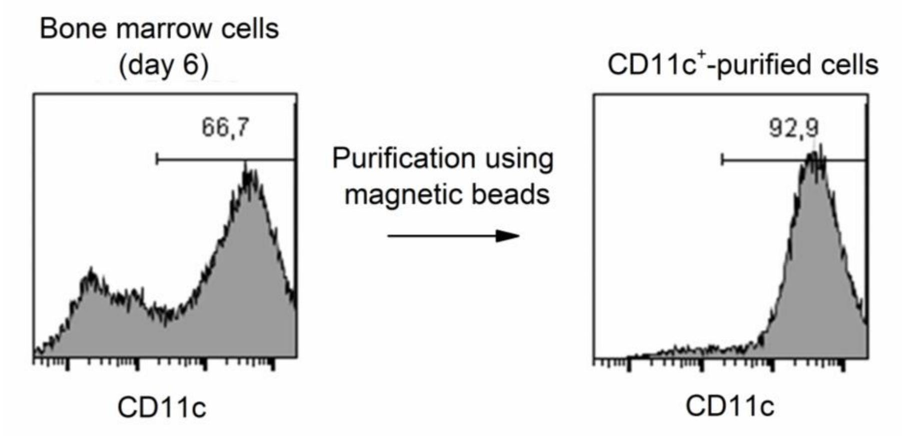


**Supplementary Figure S1.**

Percentage of CD11c+ cells in BMDC after 6 days in GM-CSF and after purification with CD11c-specific magnetic beads.

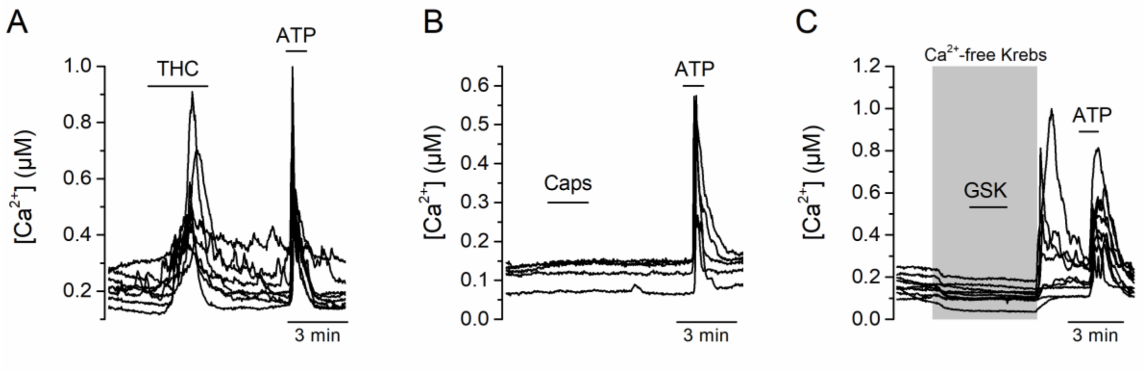


**Supplementary Figure S2**.

(**A–C**) Representative traces of intracellular $Ca^{2+}$ levels in CD11c+ BMDC stimulated with tetrahydrocannabinol (panel A, THC, 50 μM) capsaicin (panel B, Caps, 1 μM) or GSK1016792A (GSK, 3 μM) in the absence of extracellular $Ca^{2+}$ (panel C). The horizontal lines indicate the periods of application. ATP (100 μM) was applied as a positive control for intracellular $Ca^{2+}$ increase.

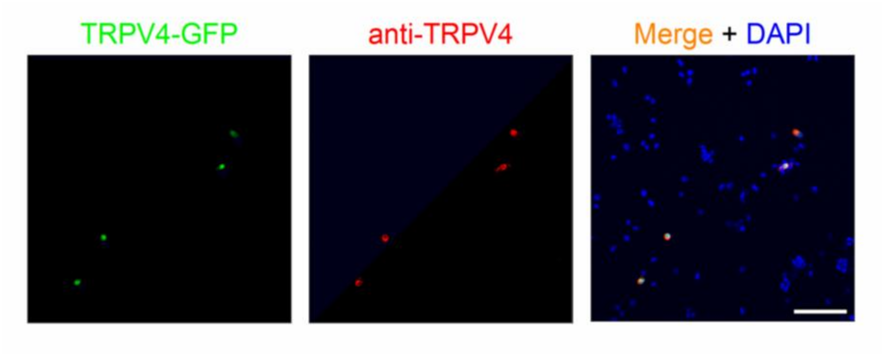


**Supplementary Figure S3**.

Confocal imaging of HEK293T cells transfected with a TRPV4-GFP construct showing the specificity of the anti-TRPV4 antibody used in Figure 1B. Transfected cells, shown in green in the left image, are also detected red after incubation with rabbit anti-TRPV4 followed by anti-rabbit Alexa 594 antibodies (middle image). Scale bar, 100 μm.

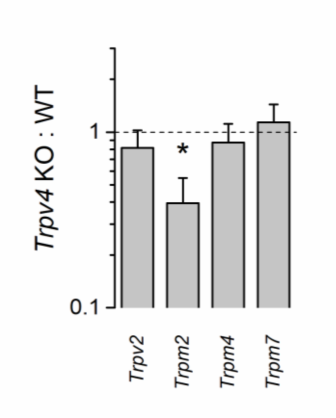


**Supplementary Figure S4**.

TRPM2 expression is downregulated in *Trpv4* KO BMDC. Relative expression levels (normalized to wild type, WT) for represented gene transcripts from *Trpv4* KO CD11c$^{+}$ BM-derived cells. *, $p < 0.05$, two-tailed, unpaired $t$ test. The data is represented as mean ± SEM of three independent experiments.

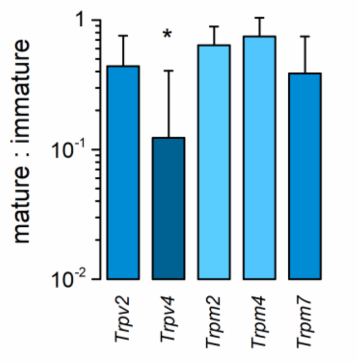


**Supplementary Figure S5.**

TRPV4 is downregulated in mature CD11c⁺ BMDC. Relative expression levels (normalized to immature cells) for represented gene transcripts in cDNA from mature CD11c⁺ BM-derived cells. *, $p < 0.05$, two-tailed, unpaired $t$ test. The data is represented as mean ± SEM of three independent experiments.

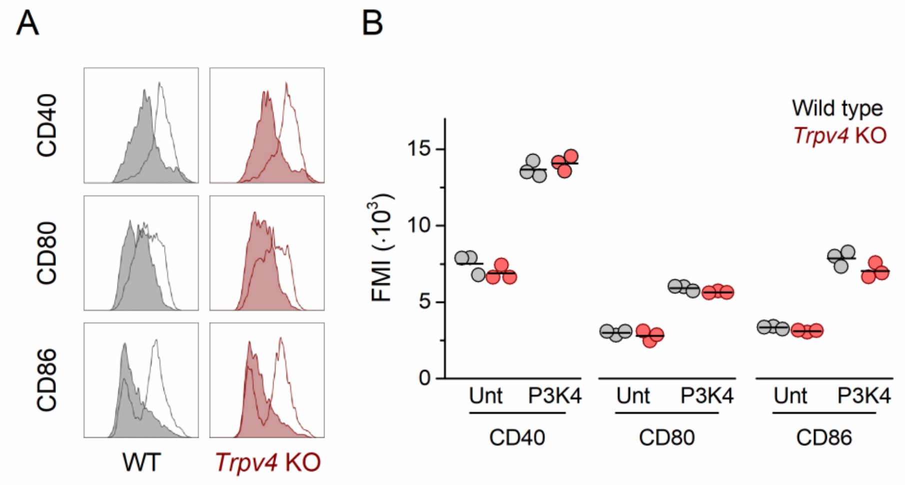


**Supplementary Figure S6.**

TRPV4 is dispensable for TLR1/TLR2-mediated maturation of CD11c⁺ BMDC. (**A**) Representative histograms of wild type (WT) and *Trpv4* KO CD11c⁺ BMDC untreated (shaded histograms) or treated (empty histograms) with TLR1/TLR2 agonist Pam3CSK4 (P3K4, 1 µg/ml). Cells were harvested after 6 days of differentiation and incubated with Pam3CSK4 for 24h. Histograms correspond to living, single cells gated within the CD11c⁺ population. (**B**) The right panel shows corresponding mean fluorescence intensity (MFI) values for CD40, CD80 and CD86 expression from WT and *Trpv4* KO CD11c⁺ BMDC untreated or treated with Pam3CSK4. Data points correspond to living, single cells gated within the CD11c⁺ population. The horizontal bar represents the mean of three independent experiments. Unt, untreated cells. P3K4, Pam3CSK4-treated cells.

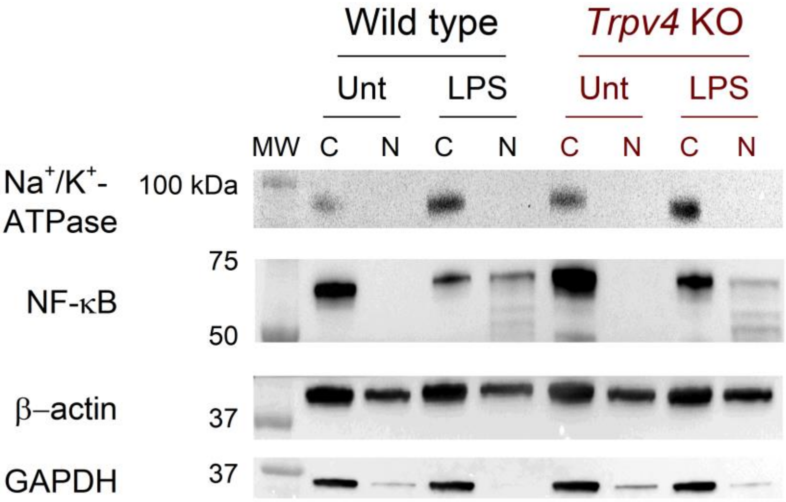


**Supplementary Figure S7**.

Western blot analysis of NF-κB p65 nuclear translocation in WT and *Trpv4* KO BMDC. Cells were untreated or treated with LPS (100 ng/ml) for 30 min. $Na^+/K^+$-ATPase and GAPDH were used as a cell fractionation control for cytosolic/membrane content. β-actin was used as a loading control for both the cytoplasmic and nuclear fraction.

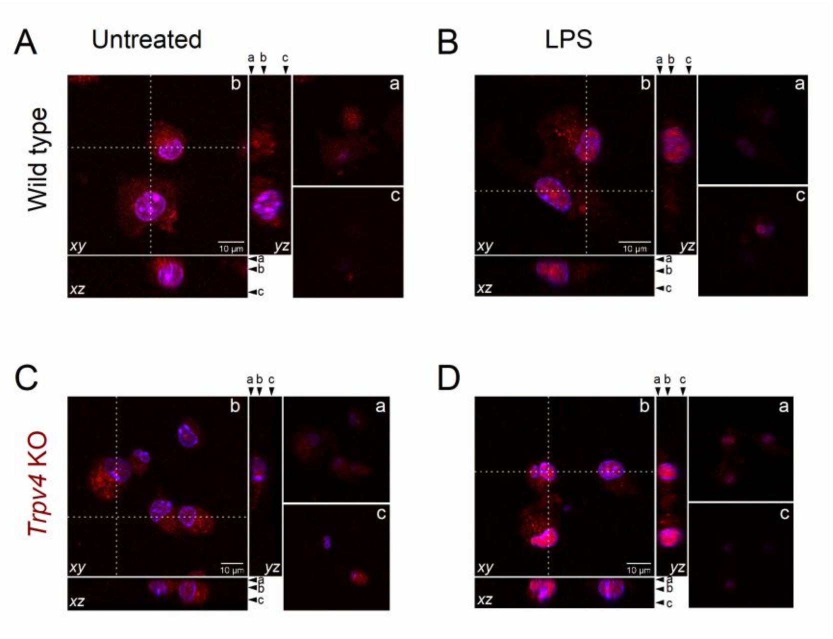


**Supplementary Figure S8**.

Representative confocal immunofluorescence microscopy images of fixed BMDC untreated or treated with LPS (100 ng/ml). Cells staining corresponds to NF-κB p65 (red) and DAPI (nuclear, blue). Scale bar, 10 µm. The selected *xy*-plane images correspond to positions on the *z*-axis located above (c) and below (a) the cell, and in the middle of the nucleus (b).

(**A**) Wild type, untreated. Stack depth $z$ = 25 frames, steps = 0.5 µm. The *xy*-plane images correspond to $z$ values of 3 (a), 12 (b) and 29 (c).

(**B**) Wild type, LPS-treated. Stack depth $z$ = 32 frames, steps = 0.5 µm. The *xy*-plane images correspond to $z$ values of 3 (a), 10 (b) and 21 (c).

(**C**) *Trpv4* KO, untreated. Stack depth $z$ = 30 frames, steps = 0.5 µm. The *xy*-plane images correspond to $z$ values of 3 (a), 10 (b) and 21 (c).

(**D**) *Trpv4* KO, LPS-treated. Stack depth $z$ = 25 frames, steps = 0.5 µm. The *xy*-plane images correspond to $z$ values of 2 (a), 10 (b) and 22 (c).

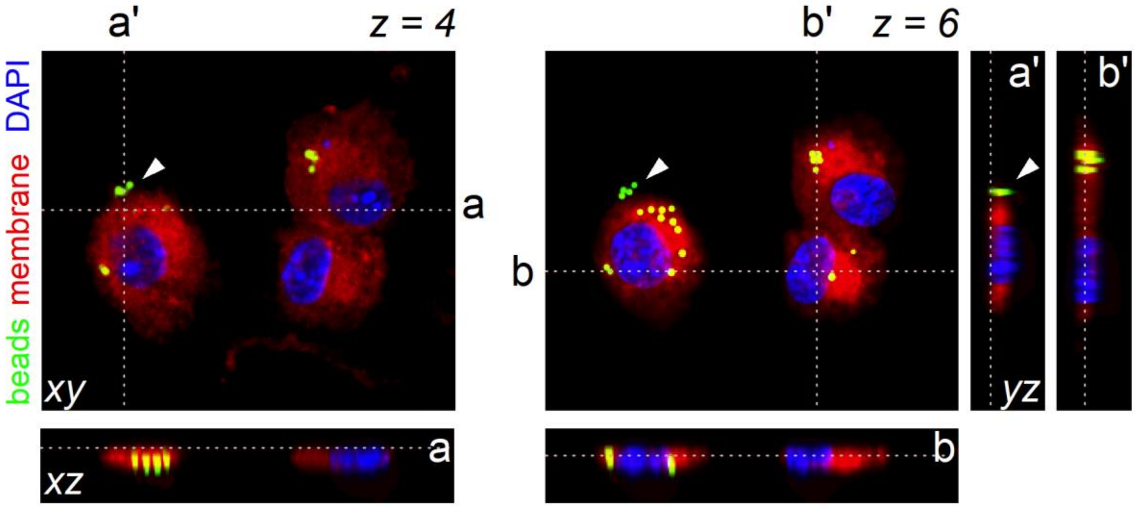


**Supplementary Figure S9**.

Representative confocal immunofluorescence microscopy images of BMDC incubated with Fluoresbrite microspheres (green) for 30 min. Cells were stained with CellMask Deep Red Plasma membrane stain (red) and DAPI (nuclear, blue). The selected *xy*-plane images correspond to positions 4 (a, a' in the orthogonal view) and 6 (b, b' in the orthogonal view) on the *z*-axis. Stack depth $z$ = 11 frames, steps = 1.18 μm. Non-internalized beads (white arrow heads) are clearly not overlapping with membrane staining (red) and therefore excluded from the quantification.
